# Best practice portals in health promotion and disease prevention: approaches, definitions, and intervention evaluation criteria

**DOI:** 10.3389/fpubh.2025.1480078

**Published:** 2025-01-28

**Authors:** Maria Piotrowicz, Małgorzata Gajewska, Katarzyna Lewtak, Ewa Urban, Anna Rutyna, Aneta Nitsch-Osuch

**Affiliations:** ^1^Department of Health Promotion and Chronic Disease Prevention, National Institute of Public Health NIH – National Research Institute, Warsaw, Poland; ^2^Department of Social Medicine and Public Health, Medical University of Warsaw, Warsaw, Poland

**Keywords:** best practice, good practice, promising practice, intervention, portal, health promotion, disease prevention, evidence-based

## Abstract

**Introduction:**

The evaluation of practices is a valuable source of evidence in the context of an evidence-based approach to public health. Best practice portals (BPPs) are promising tools for facilitating access to recommended programmes, monitoring and improving the quality of interventions. There are several such portals in Europe, but there is little work in the scientific literature on the subject. The study aimed to identify and characterise BPPs in health promotion and disease prevention and analyse the approaches, definitions, and criteria for evaluating interventions.

**Methods:**

To identify portals, websites of public health institutions and organisations, the PubMed database and grey literature were searched. The material consisted of elements of each portal’s design, information available on their websites, and collected publications. The study applied a qualitative analysis with a descriptive approach and covered a detailed description of the four selected portals.

**Results:**

Among the analysed BPPs, three were from the European region, and one was from Canada (pioneer in developing best practice tools). The dates of launching the portals ranged from the year 2003 to 2016. The number of interventions collected in the databases ranged from 120 to 337. Portals were useful, well-designed, and developed tools. BPPs differed in terms of their objectives and roles, adopted standards and criteria for assessing practices, and other operational factors. In each portal, interventions underwent a rigorous and multilevel assessment process conducted by independent experts in the field and based on intervention evaluation criteria. Generally, the analysed catalogues described similar issues, e.g., Selection of the issue addressed by the practice, Description of a particular element of the practice, Theoretical foundation, or Evaluation/Effectiveness. However, we identified both similarities and differences in the adopted terms (names of criteria) and their definitions. It was shown that sometimes the same criterion had different names depending on the catalogue. On the other hand, criteria with identical or similar names could be defined differently within the detailed thematic scope.

**Conclusion:**

The similarities and differences presented in this work can serve as a valuable starting point for designing such tools to support practice-based and evidence-based decision-making in health promotion and disease prevention.

## 1 Introduction

Over the past two decades, progress in evidence-based medicine has led to the development of evidence-based and practice-based approaches in public health. The evaluation of public health interventions (practices) is a valuable source of evidence (1, 2). In Europe, there are several portals that collect information on interventions with documented effectiveness in the field of public health, health promotion, and disease prevention. Appropriate evaluation and assessment criteria are used in selecting interventions (3). Best practice portals, also referred to as repositories or intervention registries, are a promising tool for broad strategies (local, regional, national or international) that are aimed at improving population health and its determinants through access to recommended programmes, monitoring, and improvement of the quality of public health interventions (4).

The goal of creating best practice portals is to aggregate extensive knowledge obtained from science and practice, which is currently growing significantly, and to ensure access to selected and up-to-date information on “what works” for policymakers, practitioners, researchers, and citizens. Online practice portals have been created to simplify the task of searching for evidence-based and effective interventions, which can serve as ready-to-use models or their modalities (5). Validation of an intervention that results in its inclusion on a professional and reliable portal with designation as best, good, or promising allows to make the given intervention credible and often it is helpful or required for intervention implementation and for obtaining funding from public regional or central institutions. Research shows that users of best practice portals most often use them for two reasons: selecting a new intervention to implement and validating an existing intervention (6).

The abovementioned conditions have led to an increase in the popularity of portals that collect information on practices with documented effectiveness. These portals serve important functions. However, experiences gathered in countries with a long history of using the portals show that there are many needs and challenges associated with keeping and updating the repositories (6, 7). Countries that do not yet have a national database of best practices in health promotion and disease prevention but they are planning such initiatives (e.g., Poland through the development of the ProfiBaza portal) may be interested in learning about existing solutions for designing best practice portals, as well as the benefits and challenges associated with their operation. In the scientific literature, there are few studies on detailed characteristics of best practice portals or comparative analyses of the adopted definitions of practices and the evaluation criteria (3, 8).

The objectives of the study were: (1) to identify publicly available portals/databases that collect information on health promotion and disease prevention interventions, including best, good, and promising practices; (2) to characterise selected portals/databases; and (3) to analyse the approaches, definitions, and criteria for evaluating interventions that have been used in the identified portals/databases.

## 2 Methods

To identify portals that collect information on recommended practices in health promotion and disease prevention, the following sources were searched: websites of public health institutions and organisations from European countries (public health institutes, government agencies, international organisations and other institutions) and the bibliographic database PubMed. Additionally, scientific publications and grey literature were searched using the Google Scholar search engine. Many combinations of search terms were piloted in order to find the most appropriate search algorithm. Initially, the following combinations of terms were used:

- intervention, practice, programme, service, project, policy, action, activity;
- best, good, promising, recommended, evidence-based or evidence-informed, practice-based evidence;
- criteria, assessment, evaluation, rating, quality, effectiveness, efficiency;
- database, portal, register, resources, repository;
- health promotion, disease prevention, health education, public health, lifestyle, health behaviour, disease (self)management.

Given the aim of the study, the articles had to fulfil the criteria listed below in order to be eligible for thematic synthesis:

1. present an online portal/database that systematically presents information about best, good, or promising practices;
2. focus on the field of public health, including health promotion, disease prevention, health education, and health behaviours, excluding databases with a narrow focus, i.e., those focusing exclusively on a single health issue or disease (e.g., addictions);
3. provide in English language detailed information about the portal, including the intervention evaluation process and the evaluation criteria/sub-criteria, along with their definitions.

The articles were not eligible if:

- portal/database of best, good, or promising practices was not the main or secondary topic;
- portal/database did not focus on the thematic field defined above but it focused on clinical issue(s), specific health problem(s), or disease(s);
- portal/database presented practices that were not used in Europe.

The study was limited to European portals (with one exception described below) due to the common cultural context, as well as limitations on the elaboration of analysed materials (see Limitations and strengths of the study section for explanation).

**Figure 1:**
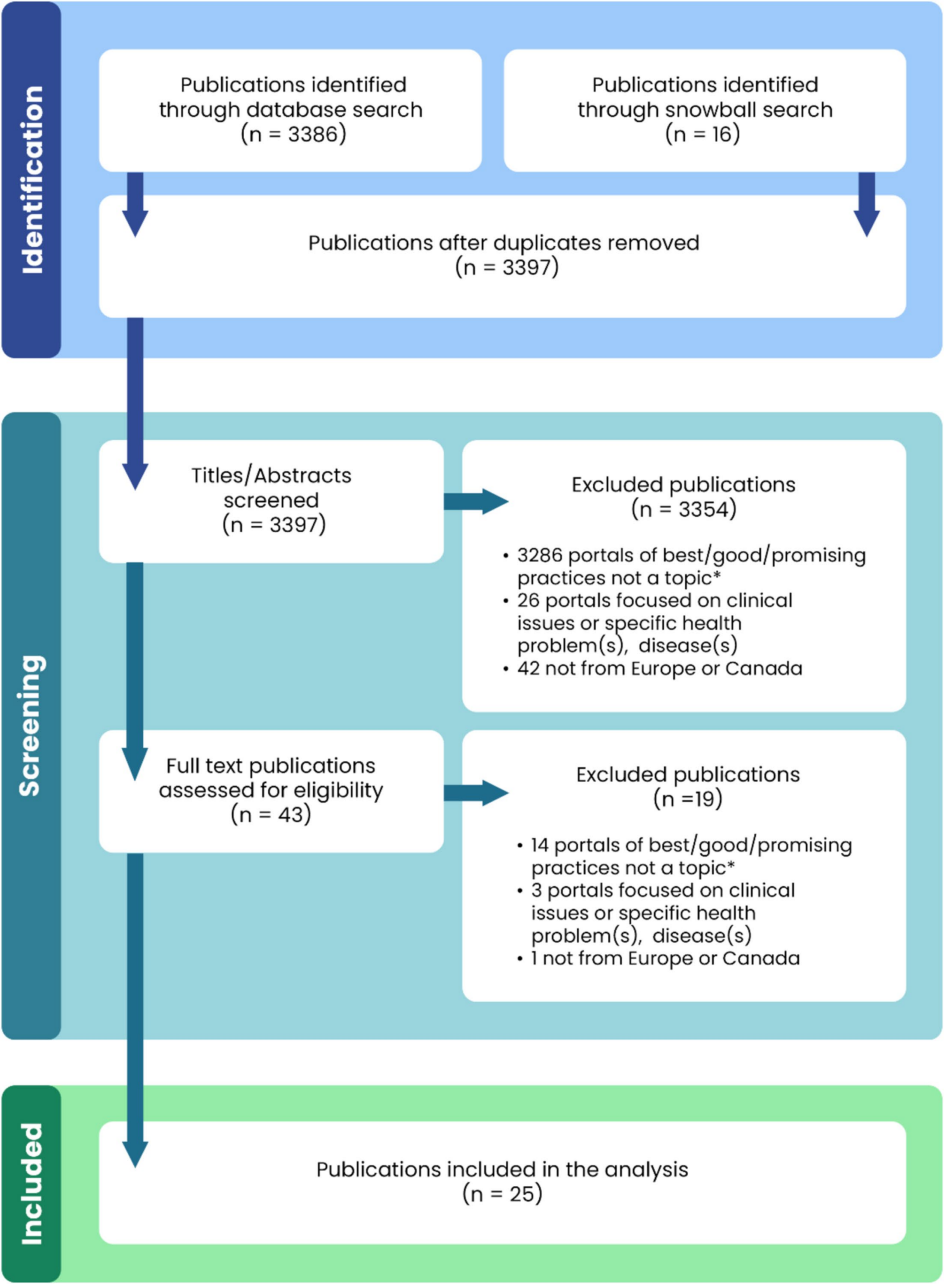
Flowchart of publication selection. *The publications did not concern a specific portal or portals of best/good/promising practices in public health, nor did they address the subject matter of portals of best/good/promising practices in public health in general. They are mainly concerned with public health or healthcare issues, including those related to good practice, but without reference to a portal/repository collecting data on numerous public health practices.

The search in the PubMed database included publications in English, with access to abstracts and free full text from 2000 to 2024. The selection of search terms sought to optimise comprehensiveness with precision. In addition, all references of the publications included in the thematic synthesis were screened and grey literature was searched, which is recommended to identify articles that would otherwise be missed through a process of snowballing. De-duplication of the search results was performed. The screening of titles and abstracts according to the eligibility criteria was performed. Full texts of all potentially relevant publications were read and rated for the final inclusion of articles. The screening process was conducted by two authors. The flowchart of publication selection is presented in Figure 1.

Detailed characteristics of the finally qualified publications are described in Appendix 1.

Finally, according to the inclusion criteria, the analysis covered four portals/databases:

- European Commission’s Public Health Best Practice Portal (EC PHBPP),
- Canadian Best Practices Portal (CBPP),
- Loket Gezond Leven/The Dutch Effectiveness Rating System (ERS),
- Praxisdatenbank Gesundheitliche Chancengleichheit, Germany (PGC).

The selection of portals was influenced by the availability of detailed information in English about the portals, particularly the availability of criteria catalogues and their definitions. The review provided such detailed information regarding three portals from Europe. The literature review also provided numerous publications that described the Canadian portal and its pioneering role in developing best practice tools. The availability of publications providing information in English about this portal (defined in the aim of the study), the long history and recognition of this database were the reasons for inclusion of the Canadian portal in the analysis.

The review also identified portals that were not included in the analyses due to the limited availability of information on these portals sought in accordance with the objectives of the study. These included Pro.Sa database (Italy), Platforma za izmenjavo dobrih praks (Slovenia) and Hyvinvointia ja terveyttä edistävien toimintamallien arviointi (Finland).

The analysed material consisted of elements of the design of each portal, information available on their websites, and collected publications, including catalogues of practice evaluation criteria (Appendix 1) developed by the entities responsible for the operation of each portal.

The study applied a qualitative analysis with a descriptive approach, which was conducted in three stages. In the first stage, a description of the portals was made, including a comparison of features such as the territorial scope of the database, launch date, number of interventions collected in the database (as of January 2024), subject area, and definitions of practices. In the next stage, the process of intervention evaluation in each database was described, including submission/screening of practices, evaluation methods, and evaluation results. The final stage of the analysis covered the catalogues of intervention evaluation criteria/sub-criteria in each database, including:

- the structure of the catalogues (i.e., the number of criteria and criterion groups, the concept of presentation/description), and
- criteria definitions (i.e., the terms/names of the criteria, their content including general meaning and detailed thematic scope, as well as assignment to a criterion level or criterion group).

Due to the significant variety of terms used in the analysed materials, in the following parts of the study, the terms portal/database and intervention/practice are used interchangeably.

## 3 Results

### 3.1 Portal/database characteristics

The detailed characteristics of individual portals are presented in Table 1. Among the analysed portals that collect data on interventions, three were from the European region, and one was from Canada. The European Commission portal (EC PHBPP) collected practices from various European countries, while the Dutch (ERS) and German (PGC) portals contained databases of interventions exclusively from their own countries. The Canadian database (CBPP) served as a repository of practices primarily from Canada and the USA, but it also included Aboriginal public health initiatives implemented in Australia.

**Table 1:** Characteristics of best practice portals in health promotion and disease prevention (Data as of January 2024).

Name of portal (territorial scope)	European Commission Public Health Best Practice Portal (EC PHBPP)^1^ (European Union)	Canadian Best Practices Portal (CBPP)^2^ (Canada, USA)	Loket Gezond Leven/The Dutch Effectiveness Rating System (ERS) for health promotion interventions^3^ (Netherlands)^4^	Praxisdatenbank Gesundheitliche Chancengleichheit (PGC) (Germany)^4^
Website	https://webgate.ec.europa.eu/dyna/bp-portal/	https://cbpp-pcpe.phac-aspc.gc.ca/	https://www.loketgezondleven.nl/	https://www.gesundheitliche-chancengleichheit.de/praxisdatenbank/
Leading/responsible entity	Directorate General for Health and Food Safety European Commission (DG SANTE)	Public Health Agency of Canada	National Institute for Public Health and the Environment in the Netherlands Original name: Rijksinstituut voor Volksgezondheid en Milieu - RIVM	Federal Centre for Health Education and German Cooperation Network ‘Equity in Health’ Original name: Bundeszentrale für gesundheitliche AufKlärung – BzgA and Kooperationsverbund Gesundheitliche Chancengleichheit
Launch date	2016	2006	2008	2003
Scope of portal (detailed subject area/field of collected interventions)	Health promotion Disease prevention Public health Management of non-communicable diseases Other areas	Health promotion Disease prevention Public health planning	Health promotion Disease prevention Lifestyle interventions People’s behaviour change and influencing their circumstances	Health promotion Addressing social determinants and socially disadvantaged groups Setting-based health promotion addressing health inequalities Equity-oriented health promotion
Types of interventions collected in the database	Action programme E-health & mHealth Health care service delivery Health in all policies Information/awareness raising campaign Intervention National health promotion programme Policy Research project/programme School based intervention Screening Tool/instrument/guideline Training Workplace intervention mHealth Other	Interventions Practices Best Practices Promising Practices Aboriginal ‘Ways Tried and True’^5^ Programmes Policies Services Strategies	Interventions Practices Programmes Approaches	Interventions Practices Good-practices Measures Projects Programmes Networks
Definitions/description of interventions collected in the database according to the classification/designation	**Best Practice:** relevant policy or intervention implemented in a real life setting which has been favourably assessed in terms of adequacy (ethics and evidence) and equity as well as effectiveness and efficiency related to process and outcomes. Other criteria are important for a successful transferability of the practice such as a clear definition of the context, sustainability, intersectorality and participation of stakeholders.	**Best Practice:** intervention, programme, or initiative that has, through multiple implementations, demonstrated: high impact (positive changes related to the desired goals), high adaptability (successful adaptation and transferability to different settings), and high quality of evidence (excellent quality of research/evaluation methodology, confirming the intervention’s high impact and adaptability evidence). Promising Practice: intervention, programme, service, or strategy that shows potential (or “promise”) for developing into a best practice. Promising practices are often in the earlier stages of implementation, and as such, do not show the high level of impact, adaptability, and quality of evidence as best practices. However, their potential is based on a strong theoretical underpinning to the intervention.	Recognised Intervention: no specific definition; requiring to meet criteria for the following recognition levels: **Effective intervention:** Proven effectiveness in theory and practice (general criteria for all the levels of effectiveness). Effective including 3 sub-levels: First indications for effectiveness Good indications for effectiveness Strong indications for effectiveness **Theoretically sound intervention:** Sound underpinning based theory, modelling of research outcomes. **Well described intervention:** Good description of goals, target group, approach, preconditions. Good starting point in quest for underpinning and evidence.	**Good Practice:** no specific definition; meeting min. 3 criteria among 12 defined criteria of good practice required
Number of interventions collected in the database according to their classification/designation (as of January 2024)	Best/Good Practices – 257	Best Practices – 37 Promising Practices – 84 Aboriginal Ways Tried and True^5^–41 Other – 119 Total – 281	Effective – 36 Theoretically sound – 153 Well described – 148 Total – 337	Good Practices – 120 Other – 3,009 Total – 3,129
Time of registering interventions in the database	2016–2022	2006–2016	2008–2023	2003–2019
Template for presenting an intervention	Origin (project type^1^) Geographical area Country Title (EN) Title (Original) Acronym Target group Type of intervention General health topic Year of selection Keyword Classification File (summary) Other	Title Categories/infographics associated with practice among following: Intervention Type (Promising Practices, Aboriginal Ways Tried and True, Best Practices) Special Characteristics (Health Equity, Canada) Population (age group, sex, other) Intervention Focus: Behaviour-Related Risk, Prevention of Diseases, Promoting Health Settings (Community Settings, Educational Settings) Determinants of Health Health Promotion Strategy Other (Language English) Overview (short description) Link to Intervention Site	Title Date of recognition Designation The intervention in brief (Target audience, Goal, Approach in brie) Performance (Type of organisation, Conditions, Materials, Documents) Quality and effectiveness (Recognition/Designation level, Date of recognition, Judgement committee, Intervention was assessed by, Research evidence) Contact (Owner name, Contact details)	Good practice designation Title Provider name Year of Publication Short description with goals and measures Contact information Project sponsor Background Objectives and target groups Continuation assumption Good practice criterion Lessons learned Literature Duration of the practice Exclusion groups Age groups Multipliers/Continuators Partners Detailed objectives Quality assessment Settings Launch date
Search filters	Advanced search (with combination of chosen sub-categories): Countries Area/Topic of interest (General health topic) Origin: Project/Joint Action Type of practice Year of selection	Advanced search (with combination of chosen sub-categories) -> The same as categories above/infographics associated with practice	Simple search: Health topics Target audiences (age, socio-economic status, health status) Educational settings Childcare settings Advanced search (with combination of chosen sub-categories) -> The same filters as in simple search plus: Sub-categories of health topics Recognition level (designation) Scope of intervention (e.g., education, legislation, change in the setting) Settings	Possibility to search through practices that meet good practice criteria or through all interventions in the database” Simple search: Settings Topics/Intervention fields Target group Age group Advanced search (with combination of chosen sub-categories) -> The same filters as in simple search plus: Gender/Sex Good practice criterion Provider or Project name Zip code/City Federal state/Geographical area Full text search and List of selected categories

1 Also include practices origin from projects: JANPA, CHRODIS (2014–2017) and CHRODIS PLUS (2017–2020).

2 The portal website is archived and publicly available. The portal was included in the analysis due to its pioneering character in terms of developing this type of tool and the availability of information on methods and criteria for intervention assessments. At the time of this writing, as of January 2024, the database is no longer available.

3 The name of the portal in scientific literature in presented in English. The abbreviated English name was used in this work (ERS).

4 Two databases, i.e., Dutch (ERS) and German (PGC), are available only in the native languages. The analyses were supported by a translator.

5 The portal includes interventions classified as Aboriginal ‘Ways Tried and True’ implemented and worked in Aboriginal contexts, which were not covered in the analyses in this paper due to their specifics, i.e., separate definitions, criteria, and assessment process.

The institutions responsible for maintaining the databases include government agencies, international organisations, or national associations/consortia of public and social entities. The dates of launching the portals ranged from the year 2003 to 2016.

The main purpose of all portals was to select and evaluate practices according to the defined evaluation criteria, and then make the good practice database available to stakeholders. With regard to the specific scope of the portals, all databases collected practices in the field of health promotion and disease prevention, but each put stress on different, specific subject areas. EC PHBPP focused on interventions aimed at combating non-communicable and communicable diseases, thus it broadly covered the field of public health. CBPP focused on chronic disease prevention and health promotion practices, ERS concentrated on lifestyle interventions, whereas PGC gathered health promotion interventions for socially disadvantaged populations.

The databases used different terminology to describe the collected practices. The most frequently applied terms that were used interchangeably were “intervention” and “practice.” The review of the types of practices identified in the databases revealed that these terms also covered the notions of programme, project, research project, measure, activity, approach, service, strategy, policy, guideline, and network.

Practices collected in the databases were given various names and definitions. The most common terms were best, good, or promising practice. However, the ERS used the term “recognized intervention” to avoid confusion because various definitions of best, good, or promising interventions exist (9). Nevertheless, practices collected in each of the presented databases were subject to similar evaluation standards (3). The definitions referred directly to the adopted assessment criteria on a given portal. In the case of two portals, definitions were presented for particular designations (EC PHBPP, CBPP), listing the key assessment criteria. The other databases did not introduce separate definitions for particular designations but described them through adopted catalogues of assessment criteria (ERS, PGC).

The number of interventions collected in the databases ranged from 120 to 337, depending on the portal (as of January 2024). In two databases (CBPP, PGC), best, good, or promising practices were subsets of all the interventions listed in the databases.

The length of the period when interventions were recorded in the databases varied depending on the portal. The longest periods were observed on PGC and ERS portals, which have been collecting practices since 2003 and 2008, respectively. The number of practices in the databases changed dynamically, and it was related to both newly added practices and the “expiration date” of already evaluated interventions. Interventions were subject to re-evaluation after a defined period (details are described in the next section of the Results).

In the analysed databases, each intervention was presented in a structured form of a summary of the characteristics of the intervention. This presentation allowed for a quick and accessible review of typically extensive information about a specific intervention. Commonly used elements of the presentation included issue date, practice title, designation in the database, summary, health topic, owner and contact details. Templates also included a downloadable file with a detailed description of the intervention or a link directing to a webpage.

A common feature of the described databases and a useful tool for using them was a simple and/or advanced search function for selecting interventions. Searching was offered through several filters, usually with many sub-levels (ranging from several dozen to over a hundred) and keywords. The most commonly used searching filters were health topic, setting and target group.

### 3.2 Intervention evaluation process

#### 3.2.1 Submission or screening of practices

In two of the analysed databases (EC PHBPP and ERS), the process of selecting practices for evaluation involved accepting submissions from those who own the intervention (4, 9). Owners could be various entities such as national, regional, or local organisations, non-governmental organisations, or even citizens. In addition to the usual submission process throughout the year, interventions were also sent to the EC PHBPP portal in response to calls for submissions, which are commonly announced on the portal.

Interventions from the government, research, and non-profit organisations, and research databases were screened on CBPP by public health researchers (10, 11). A different approach was presented on the PGC, where only those interventions were submitted for evaluation that had previously been recommended by the Coordination Office of Equity in Health (3).

Owners of the evaluated interventions were required to submit detailed descriptions of the practice along with all materials and responses needed for successful evaluation. Special online questionnaires for this purpose were made available on the EC PHBPP, ERS, and PGC websites (12–14). At each stage of submitting an intervention for evaluation, owners could receive support offered by the portal experts. For example, ERS provided advice on draft descriptions by external advisors to improve the quality of submissions, while the PGC database involved in-depth interviews with practice owners.

#### 3.2.2 Evaluation process and its result

In each database, interventions underwent a rigorous assessment (evaluation) conducted by internal and/or external independent experts in the field. The assessment was always a multilevel process, and inclusion and/or exclusion criteria were considered. At this stage, submitters could be asked for revision and additional information. The process of evaluating interventions varied slightly in each database. The result was either the inclusion of the intervention in the database with a designated category (Table 1) or the rejection of the intervention if it did not meet the requirements.

In the first stage, interventions on the EC PHBPP were evaluated by three experts: two external evaluators and one internal (4, 15). The evaluation of interventions began with the exclusion criteria. Reviewers then prepared a scoring assessment for the preliminarily qualified interventions. Only those interventions that scored at least 328 points (i.e., 68% of the maximum possible score) were considered “best practices.” The final decision on classifying an intervention as a “best practice” was made by the Directorate General for Health and Food Safety. Interventions were evaluated once a year, and the entire evaluation process took approximately 6 months. Additionally, the database included practices that were collected and transmitted through actions co-funded under the EU Health Programmes in the previous 5 years.

On the CBPP, interventions were evaluated by a team of experts in the following steps: assessment quality of evaluation or study design, search for additional information on selected individual interventions, expert review using inclusion criteria, prioritisation of selected interventions for annotation, and selection of resources (10, 16). All interventions recognised as “best practices” had a validity period of 10 years. For “promising practices,” the validity period was 5 years, unless the practice, in light of new data, was reclassified as a “best practice.”

In the ERS, two evaluation pathways were distinguished depending on the designation level/category sought by the submitter (9, 17). In the first pathway, the practice was evaluated by the relevant recognition committee with representatives from science, practice, and policy. In this pathway, the final decision on including the intervention in the database was associated with assigning one of the following designations: “Theoretically Sound” or “Effective” (including 3 sub-levels) (Table 1). The recognition committee held approximately eight meetings per year. In the second pathway, the intervention was evaluated by assessors from the field, and it could receive the designation “Well Described.” This type of assessment was made three times a year.

In order to continuously improve the quality of interventions listed in the database, after 3 years (“Well Described” interventions) or 5 years (“Theoretically Sound” or “Effective” interventions), the intervention owner received an invitation for re-evaluation. If specific criteria were met, a “Well Described” intervention could be upgraded to a “Well-Substantiated” intervention.

In the PGC, interventions positively assessed by the Coordination Office of Equity in Health were designated as “good practices.” These practices had to meet 3 out of 12 assessment criteria to be considered by reviewers (3). There was no exclusion of practices from the German register if they failed to meet the assessment criteria, as the adopted concept was that “the point is not to distinguish criterion implemented from criterion not implemented, but to reach a higher level of quality step by step” (18).

### 3.3 Catalogues of assessment criteria and sub-criteria

The analysis of the criteria catalogues used to assess interventions collected in the databases revealed both similarities and differences in terms of the structure of the catalogues and the definitions of the criteria.

#### 3.3.1 Structure of the catalogues

**Figure 2:**
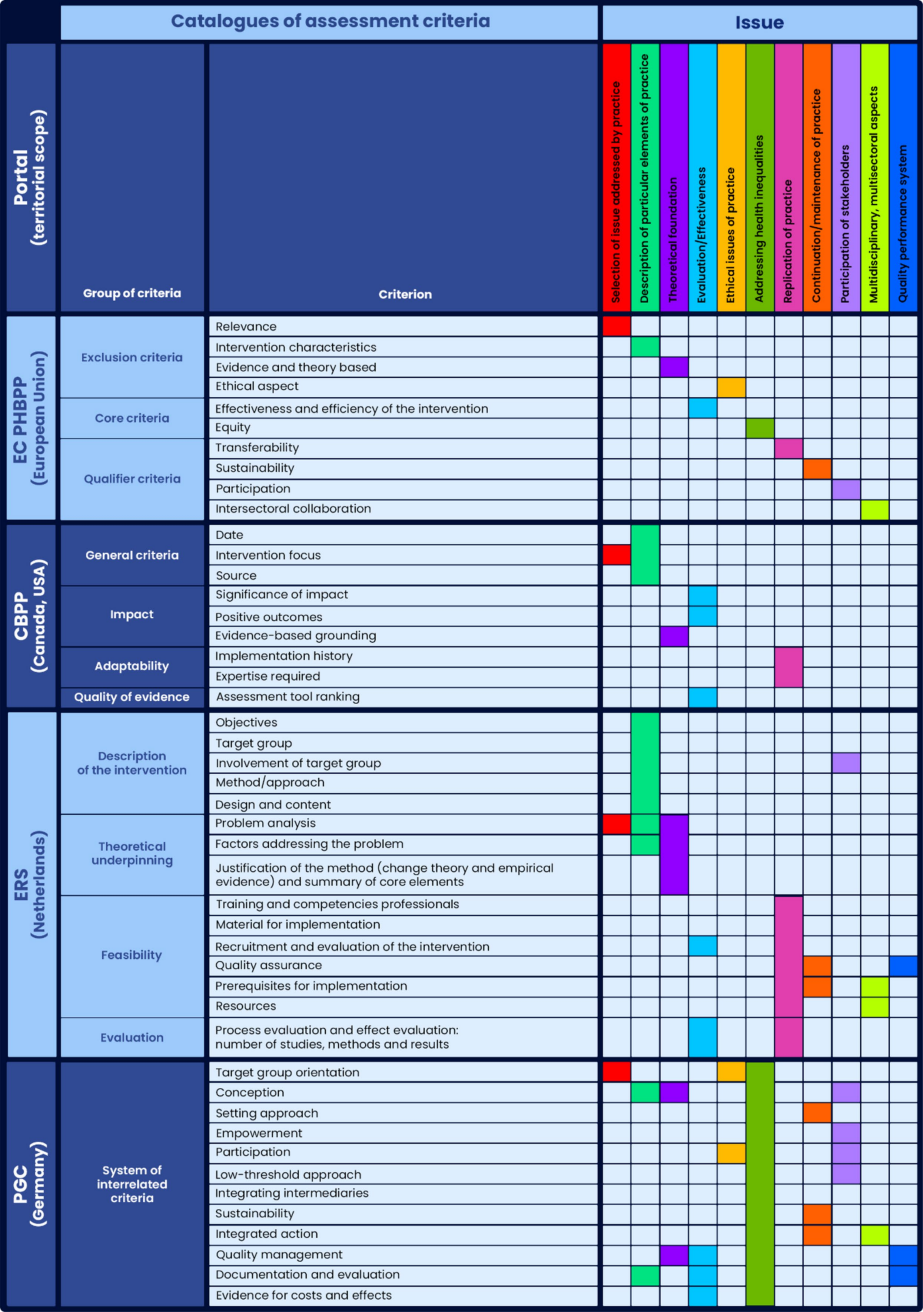
Common issues for assessment criteria catalogues identified in the qualitative analysis of criterion’s content* (general meaning and detailed thematic scope). *The merged cells refer to the entire group of criteria.

Each database had its own catalogue, characterised by a specific structure (Figure 2). The catalogues provided detailed definitions for each of the adopted criteria. The EC PHBPP catalogue contained 10 criteria divided into three groups (Exclusion, Core, and Qualifier criteria). Each criterion was defined descriptively, using between 2 and 9 sub-criteria. The catalogue of criteria was available as guidelines/instructions for submitters (19, 20). The European Commission recognised the need to revise the current set of criteria (21). The update work also included defining the criteria for “promising practices” based on best practice criteria, but meeting the minimum requirements for this new type of practice. The final results of this work have not yet been published.

On CBPP, interventions were assessed based on nine criteria divided into four groups: General Criteria, Impact, Adaptability, and Quality of Evidence. Among these criteria, three were the same for both best practices and promising practices, while the remaining six criteria differed in their detailed definitions.

The source of the definitions was a publication (16), which presented each criterion descriptively. When a brief description was insufficient, the catalogue included references to other documents.

In the ERS catalogue 15 criteria were adopted, divided into four groups: Description of the Intervention, Theoretical Underpinning, Feasibility, and Evaluation. Depending on the recognition level (designation) that the submitter chose to apply for, different groups of criteria had to be met. Each criterion was defined using numerous sub-criteria. The definitions were sourced from publications and current guidelines/instructions for submitters, available only in the native language (3, 22, 23).

In the PGC catalogue, 12 assessment criteria were adopted without being grouped into categories. Each criterion was defined according to a structured description: definition, implementation levels, explanation of the levels with examples, and further reading. The descriptions also included diagrams and/or tables to facilitate understanding of particular levels of implementation or other elements of the definitions. Links between different criteria were marked in the description of each criterion. The catalogue of criteria was available on the portal in the native language and English (18).

#### 3.3.2 Criteria/sub-criteria definitions

Comparative analyses of the adopted names and definitions of criteria identified 11 issues that were present in the discussed catalogues (Figure 2). The most common issues were Selection of the issue addressed by the practice, Description of a particular element of the practice, Theoretical foundation, and Evaluation/Effectiveness. The remaining issues were less common or were less frequently identified as specific criteria. For example, ‘Addressing health inequalities’ was included as a criterion only in the EC PHBPP catalogue. This issue was not a criterion in the CBPP catalogue. However, practices that demonstrated positive outcomes among people living in conditions of disadvantage were additionally labelled as Equity-sensitive on that portal (24). The PGC database was entirely dedicated to practices targeting disadvantaged populations, so the issue of health inequalities was relevant to all criteria.

The analyses revealed that criteria with similar terminology in analysed catalogues (i.e., identical or similar names) typically addressed similar issues (in general meaning). For example, ‘Continuation/maintenance of practice’ in the EC PHBPP and PGC catalogues was expressed through a criterion with the same name: ‘Sustainability’. Meanwhile, the issue of ‘Theoretical foundation’ was expressed through criteria with similar names, namely ‘Evidence and theory based’ in the EC PHBPP catalogue and ‘Evidence-based grounding’ in the CBPP catalogue (Figure 2).

However, there were also examples of the catalogues using different criteria names to describe similar issues. A good example is the issue of ‘Replication of practice,’ which was expressed as a criterion called ‘Transferability’ in the EC PHBPP catalogue, a group of criteria called ‘Adaptability’ in the CBPP catalogue, and an element of the content of criteria in the ‘Feasibility’ and ‘Evaluation’ groups in the ERS catalogue (Figure 2).

Another aspect of the analysis showed that criteria describing similar issues (in general meaning) could differ in terms of the detailed thematic scope of the criterion’s content. The definition of a particular criterion in one catalogue did not fully correspond to its ‘equivalent’ in another catalogue.

A detailed analysis of the content of the catalogues (16, 18, 19, 23) revealed that, for example, the issue of ‘Evaluation/Effectiveness’ in all analysed catalogues referred to the assessment of interventions, and it was associated with the measurement and presentation of evidence on their effectiveness. However, a process evaluation was differentiated from outcome evaluation in the EC PHBPP and ERS catalogues, whereas the PGC catalogue introduced relevant terms: formative evaluation and summative evaluation. In the ERS catalogue, evaluation was based on the concept of the so-called ‘effectiveness ladder’ (7). This concept detailed the requirements for each level of evidence, including the number, quality, and nature of the studies supporting the intervention. In Canada, the evaluation concept was based on empirical research results with the consideration of the hierarchy of evidence for quantitative/qualitative research (25).

The greatest variation, both in terms of criteria names and their detailed definitions, was related to the issue of ‘Selection of issue addressed by practice’. In the EC PHBPP catalogue, the first criterion was ‘Relevance’, which refers to the political or strategic context of the practice and it should include information on whether it is a priority public health area, a strategy, or a response to an identified problem at the local, regional, national, or European level (19). In the CBPP catalogue, the criterion ‘Intervention Focus’ provided general guidelines: ‘Intervention must address health at a population level and can include interventions at single or multiple levels including individual, community, organisation, and societal levels. Clinical interventions are excluded’ (16). In the ERS catalogue, the criterion ‘Problem Analysis’ included content highlighting the importance of ‘the nature, severity, size, spread, perception of those involved, costs, and other possible consequences of the problem, risk, or theme’ (23). In the PGC catalogue, the starting point was the selection and characteristics of the target population, expressed in the opening criterion of the catalogue, namely ‘Target Group Orientation’ (18). The abovementioned criteria in each catalogue were not explicitly defined as determining the selection of the issue addressed by the practice. However, their position in each catalogue (usually at the beginning of the list) and their content clearly indicated their significance in the discussed matter.

In the comparative analyses of the catalogues, a challenge was the lack of consistency in assigning a particular issue to the level of a single criterion or a group of criteria. For example, the issue of ‘Theoretical foundation’ was expressed as a single criterion in the EC PHBPP and CBPP catalogues (‘Evidence and theory based’ and ‘Evidence-based grounding’, respectively). In the ERS catalogue, this issue was related to an entire group of criteria, called ‘Theoretical underpinning,’ which included three criteria. This situation was observed for other issues as well (Figure 2). Generally, the number of criteria addressing a particular issue varied across the catalogues.

## 4 Discussion

Although similar solutions are used in creating various best practice portals, they show diversity in terms of detailed portal design, intervention assessment procedures, and criteria definitions. The characteristics of the portals showed that these portals are examples of well-designed, developed tools tailored to the needs of different user groups, e.g., through standardised templates for presenting practices, availability of numerous filters, and simple and advanced search functions (Table 1). These types of functionalities increase the actual usability of the databases (6).

The websites of individual portals contained information such as the subject area of submitted interventions or definitions related to the designation of practices. However, this information was often dispersed, presented across several sections on the website, or available as downloadable instructions/guides in separate files. The preparation of materials for this work required using various sources, including information from numerous publications (Appendix 1). This could be a very demanding task for users seeking examples of good practices. Access to such information is crucial for users to understand what set of interventions has been collected in the database. Especially since, as shown in the Results of the work, the definition of practices collected on the portals is based on the adopted assessment criteria for evaluating interventions. This is related to the issue of transparency (5), which is discussed further in the paper.

Following the adopted assumptions and criteria, the results regarding the process of assessing interventions in the analysed databases show that evaluation is always carried out by a team or teams of experts in the relevant fields. The process is cyclical, quantitative and qualitative, and it is not designed for the rapid assessment of numerous practices, as it cannot be simply automated. It is typically accompanied by a dialogue and information exchange between submitters and reviewers/experts. Ultimately, the decisions that are made are the result of multi-stage agreements. Regardless of the specifics of the organisational and procedural solutions, assessment always requires consensus on assumptions and definitions, as well as human, time, and financial resources.

The analyses show that each portal has developed its own catalogue structures and definitions of criteria/sub-criteria for the assessment of practices. Typically, these are not closed catalogues, and assessed interventions do not have to meet all defined requirements perfectly. This is related to the need to evaluate very different types of interventions, ranging from relatively simple practices (e.g., school education) to extremely complex practices (e.g., national health policies) (5, 7).

Both similarities and differences in terms of the adopted names of criteria and their definitions were characterised in the analysed catalogues. It was shown that sometimes the same criterion had different names depending on the catalogue. There were also identical or similar names of criteria in the analysed catalogues. However, criteria with identical or similar names could be defined differently within the detailed thematic scope. An example is the ‘Evaluation/Effectiveness’ issue presented in Figure 2 and described in the Results section. The detailed definition of this criterion in one catalogue did not fully correspond to its ‘equivalent’ in others catalogues.

Generally, although the catalogues described similar issues (Figure 2), the abovementioned differences are worth noticing. A study from the US (6, 26) that compared evidence-based programme registers in behavioural health demonstrated that even subtle differences in assumptions, approaches, and definitions determined whether a programme was included for evaluation in specific registers and what rating/designation it received.

Portals that collect evidence-based practices, even within the same field, differ from one another due to their objectives and roles, adopted standards and criteria for assessing practices, and other operational factors. Therefore, transparency and access to the aforementioned information about the portal are particularly important from the perspective of the portal’s utility and user needs. Furthermore, defined and clear standards allow comparability and reliability of best practice repositories, which can foster easier and more rigorous exchange of best practices at national and international levels in the continent.

In the light of the analyses, it should be emphasised that the differences between the described portals from various countries/regions are influenced by the socio-cultural context, available resources, and specific needs in the area of intervention assessment that are unique to each database. In order to draw best practices, diversity is advantageous as it provides a range of example solutions for building a best practice portal.

The multiplicity of national databases (even if they are not strictly dedicated to best practices) and various requirements/guidelines from different public institutions that are aimed at health promotion practitioners can be problematic (7). In many European countries, practitioners and intervention owners must navigate through all often differing requirements, demonstrate the effectiveness of their interventions (often meeting research standards), while typically facing low priority and limited budgets for health promotion and disease prevention (27).

In addition to the numerous benefits associated with the functioning of portals, there are also limitations. Awareness of these limitations, further research, and dialogue between portal creators and users are recommended to develop more reflexive and responsive solutions. A study from the Netherlands (7) highlighted the need to monitor the functioning of the portal in the context of its intended role. Challenges were observed in stimulating the improvement of interventions. For example, the lack of consensus among various stakeholders on how the effects of certain interventions should be measured results in difficulties in assessing their effectiveness, as well as in improving them in this respect. Limitations were also related to the improvement of health promotion in practice, including issues related to the transfer of interventions and neglection of local adaptation, which can invalidate the effectiveness of evidence.

The difficulties raised in the literature concern the requirements for evaluative research supporting interventions. These requirements are often challenging to meet; for example, in the ERS, two studies that confirm effectiveness are required, while the intervention budget often covers only one study. More broadly speaking, portals are criticised for their scientific regime or fundamentally academic approach in defining assessment criteria and evaluation procedures (7, 16).

Similar challenges have been highlighted by the aforementioned American studies (6, 26), which suggest that the areas that require particular attention include the lack of research on the needs of different user groups and the actual usability of the portals. In this context, it is recommended to improve the transparency of registries, in terms of the clear definition of the purpose, scope, and assessment criteria for inclusion and evaluation processes. This is crucial because all these factors determine the content of the database. Ensuring transparency in this area can help users understand the strengths and limitations of the general approach of a particular database. The main challenges include funding and resource limitations, which primarily hinder the provision of up-to-date reviews and their timely presentation on the portal. It has been emphasised that the entire process is often labour-intensive, complex, and requires a large group of different experts.

In conclusion, the described portals have both their strengths and weaknesses (Table 2). Although the challenges outlined above are due to accumulated experience, they should not discourage, as monitoring the quality of practices and minimising the risk of implementing ineffective and sometimes even harmful interventions is crucial for development in the field of health promotion and disease prevention. The main benefit of having a national database is to advance theory and practice by consolidating experts in the field, reaching consensus and standardising basic assumptions and definitions at the national level.

**Table 2:** Strengths and weaknesses of analysed best practice portals in health promotion and disease prevention in the study.

Name of portal (territorial scope)	European Commission Public Health Best Practice Portal (EC PHBPP) (European Union)	Canadian Best Practices Portal (CBPP) (Canada, USA)	Loket Gezond Leven/The Dutch Effectiveness Rating System (ERS) for health promotion interventions (Netherlands)	Praxisdatenbank Gesundheitliche Chancengleichheit (PGC) (Germany)
Strengths of the portal:	Collecting practices from many countries that enable the exchange of knowledge and experiences at an international level. The most up-to-date database of good practices among the analysed portals. Portal in the process of development (e.g., adding new practices to the database, introducing designation - promising practice).	The pioneering character of the portal, the creation and development of the portal described in the scientific literature (Appendix 1). Distinguishing between best and promising practices (designations), which creates a database of both rigorously evaluated interventions and those with potential for further development. User-friendly portal design (i.e., clear and intuitive design through graphics describing practices).	A national database of interventions that enables comparability of good practices and exchange of experiences between stakeholders in a national context. The rating system (with 3 levels) in evaluating practices to stimulate intervention improvement. User-friendly portal design (e.g., easy navigation through the portal, and a clear template for presenting an intervention in the database).	A unique repository of health promotion practices targeting socially disadvantaged groups and reducing social inequities in health. A national database of interventions that enables comparability of good practices and exchange of experiences between stakeholders in a national context. Numerous filters for searching the database, including an interesting solution regarding the filter with a list of intervention evaluation criteria.
Weaknesses of the portal:	The collection of practices from different countries (often from a different cultural context) in a one base requires caution in making comparisons in, for example, the effects of interventions or other aspects. Template for presenting an intervention is not extensive, and could provide more information about the practice.	Strongly academic approach to evaluating interventions, requirements difficult to meet by practitioners alone, without research support. No further development of the portal (currently in archived version).	Strongly academic approach to evaluating interventions, requirements difficult to meet by practitioners alone, without research support. Non-transparent pathway for developing assessed interventions (those already presented in the database) through subsequent recognition levels.	A broad collection of interventions and good practices state a subset of all the interventions listed in the databases which can make the portal difficult to navigate. A small number of new interventions in the database in the last few years.

## 5 Limitations and strengths of the study

The study involved qualitative analyses of only four best practice portals in the field of health promotion and disease prevention. Although various European countries have similar databases, the selection of portals was influenced by the availability of detailed information in English about the portals (particularly the availability of criteria catalogues and their definitions), the history and recognition of the database. Portals with a long history were primarily selected. For these reasons, the Canadian portal was included in the study. The extensive scope of qualitative analyses justifies the smaller number of portals. A larger number of portals would have required a more focused scope of research. Another limitation is related to the fact that the study presents data on portals as of January 2024, however portal maintenance and development is a dynamic process over time. The strength of this work is that, to the best of our knowledge, it is the first study comparing various criteria catalogues for assessing best practices and their definitions. The results of this study can serve as a starting point or a matrix for further research on the collection, evaluation methods, and dissemination of interventions through portals.

## 6 Conclusion

Design, development, and launch of a portal that collects effective and recommended practices is an extremely complex undertaking, which can take several years and requires the involvement of experts from various fields, including policymakers, researchers, practitioners, managers, administrators, and IT specialists. It is essential to prepare substantive assumptions and definitions and to reach a consensus by all stakeholders.

The similarities and differences presented in this work with regard to the characteristics of best practice portals, and the process and criteria of intervention evaluation can serve as a valuable starting point for designing such tools to support evidence-based decision-making in health promotion and disease prevention. A well-conducted planning stage, which includes an understanding of the previously applied solutions in various countries, acquisition of appropriate human and financial resources, and gaining awareness of the strengths and limitations of existing portals, should precede the implementation and monitoring of a portal, considering a national setting and needs.

## Data Availability

The original contributions presented in the study are included in the article/Supplementary material, further inquiries can be directed to the corresponding author.
