# Supplementary material for: Best practice portals in health promotion and disease prevention: approaches, definitions, and intervention evaluation criteria
Source: Front Public Health. 2025 Jan 28;13:1480078. doi: 10.3389/fpubh.2025.1480078 (PMC11810953; doi:10.3389/fpubh.2025.1480078)
Supplement: Supplementary file 1 [file Table_1.docx]

Appendix 1. Characteristics of included publications (searched from form PubMed database^1^ and grey literature)

| **No.** | **Author(s) / Institution / Year of publication** | **Publication title** | **Portal / Database**^2^ | **Source of portal identification, characteristic & process assessment** | **Source of criteria definitions** | **Type of publication** |
| --- | --- | --- | --- | --- | --- | --- |
| 1. | Vinko M. et al. (2024) | Evaluator’s alignment as an important indicator of adequacy of the criteria and assessment procedure for recognizing the good practice in public health | In general,  EC PHBPP,  Other (Slovenia) | Yes | No | journal article – Original Research |
| 2. | Rossmann C. et al. (2023) | Health promotion and disease prevention registries in the EU: a cross country comparison | EC PHBPP, ERS,  PGC | Yes | Yes | journal article –  Research |
| 3. | Stepien M. et al. (2022) | European public health best practice portal - process and criteria for best practice assessment | EC PHBPP | Yes | Yes | journal article –  Methodology |
| 4. | Mielck A. et al. (2018) | German cooperation-network ‘equity in health’-health promotion in settings | PGC | Yes | Yes | journal article – Debate |
| 5. | Fazal N. et al. (2017) | Between worst and best: developing criteria to identify promising practices in health promotion and disease prevention for the Canadian Best Practices Portal | CBPP | No | Yes | journal article –  Research, Policy and Practice |
| 6. | Kok MO. et al. (2017) | Improving health promotion through central rating of interventions: the need for Responsive Guidance | ERS | Yes | Yes | journal article –  Research |
| 7. | Ng E. et al. (2015) | Framework for selecting best practices in public health: a systematic literature review | In general,  CBPP | Yes | No | journal article –  Review |
| 8. | Faggiano F. et al. (2014) | Europe Needs a Central, Transparent, and Evidence-Based Approval Process for Behavioural Prevention Interventions | In general,  ERS | Yes | No | journal article –  Policy Forum |
| 9. | Jennifer Yost J. et al. (2014) | Tools to support evidence-informed public health decision making | In general,  CBPP | Yes | No | journal article –  Research Article |
| 10. | Quinn E. et al. (2014) | How can knowledge exchange portals assist in knowledge management for evidence-informed decision making in public health? | In general,  CBPP | Yes | No | journal article –  Research Article |
| 11. | Sims-Jones N., Dyke E. (2013) | Enhancing the Canadian Best Practices Portal | CBPP | Yes | No | journal article –  Status Report |
| 12. | Finkle-Perazzo D., Jetha N. (2011) | Online resources to enhance decision-making in public health | CBPP | Yes | No | journal article –  Cross-Canada Forum |
| 13. | Brug J. et al. (2010) | Towards evidence-based, quality-controlled health promotion: the Dutch recognition system for health promotion interventions | ERS | Yes | Yes | journal article –  Point of View |
| 14. | Jetha N. et al. (2008) | Supporting Knowledge into Action:  The Canadian Best Practices Initiative for Health Promotion and Chronic Disease Prevention | CBPP | Yes | No | journal article –  Original Paper |
|  |  |  |  |  |  |  |
| 15. | German Collaborative Network for Equity in Health, Berlin (2023) | Criteria for Good Practice in Health Promotion Addressing Social Determinants developed by the German Collaborative Network for Equity in Health 1st edition, May 2023 (translation based on German edition, 4th edition, July 2021). | PGC | No | Yes | grey literature |
| 16. | Flink I. et al.  EUHealthSupport consortium, European Commission (Brussels, 2023) | Review of the EU Best Practice assessment process and portal | EC PHBPP | Yes | Yes | grey literature |
| 17. | European Commission Public Health Best Practice Portal website (Accessed January 14, 2024) | Submitter's guide Best Practice Portal | EC PHBPP | Yes | Yes | grey literature |
| 18. | European Commission Directorate-General For Health And Food Safety (Accessed January 14, 2024) | Criteria To Select Best Practices In Health Promotion And Disease Prevention And Management In Europe - Updated Version | EC PHBPP | No | Yes | grey literature |
| 19. | European Commission Directorate-General For Health And Food Safety (2022) | Minutes. Webinar on best practice review - criteria for best and promising practices (12 May 2022) | EC PHBPP | Yes | Yes | grey literature |
| 20. | MOVISIE, Netherlands Centre Youth Health (NCJ),  Netherlands Institute for Sport and Physical Activity (NISB),  Netherlands Youth Institute (NJi),  RIVM (National Institute for Public Health and the Environment), 2013 | Dutch Recognition System for Interventions. Criteria for joint quality assessment 2013-2018 | ERS | Yes | Yes | grey literature |
| 21. | van Dale D., Hendriksen M.  National Institute for Public Health and the Environment. Ministry of Health, Welfare and Sport (Accessed January 20, 2024) | Dutch Recognition System for (health promotion) interventions | ERS | Yes | No | grey literature |
| 22. | Tamsma N. et al.  World Health Organization (2018) | Good Practice Brief. Centre For Healthy Living In The Netherlands: Building sustainable capacity and alliances for effective health promotion. | ERS | Yes | No | grey literature |
| 23. | National Collaborating Centre for Methods and Tools (2010). Hamilton, ON: McMaster University (Updated 03 October, 2017) | Effective interventions: The Canadian Best Practices Portal | CBPP | Yes | No | grey literature |
| 24. | Jackson SF. et al. (2016) | A Hierarchy of Evidence: Which Intervention Has the Strongest Evidence of Effectiveness? | CBPP | No | Yes | grey literature |
| 25. | German Cooperation Network ‘Equity in Health’), Berlin (2015) | Criteria for good practice in health promotion addressing social determinants. Developed by the German Cooperation Network ‘Equity in Health‘. | PGC | No | Yes | grey literature |

^1^ The following final combination of search terms was entered into the PubMed database:

(("best practice*"[Title]) OR ("good practice*"[Title]) OR ("promising practice*"[Title]) OR (intervention*[Title]) OR ("health promotion"[Title]) OR ("disease prevention"[Title]) OR ("health behavior*"[Title]) OR ("public health"[Title]) OR ("health education"[Title]) OR ("inequalities"[Title])) AND ((portal*[Title]) OR (registr*[Title]) OR (criteria[Title]) OR (database*[Title])OR (evidence-based[Title]) OR (evidence-informed[Title]) OR (practice-based [Title]) OR (resource*[Title]) OR (rating*[Title]) OR ("literature review*"[Title]))

^2^ Portal/Database name abbreviations: EC PHBPP - European Commission’s Public Health Best Practice Portal, CBPP - Canadian Best Practices Portal, ERS - The Dutch Effectiveness Rating System (Original name: Loket Gezond Leven), PGC - Praxisdatenbank Gesundheitliche Chancengleichheit (Germany).
